# Speckle-Tracking and Tissue-Doppler Stress Echocardiography in Arterial Hypertension: A Sensitive Tool for Detection of Subclinical LV Impairment

**DOI:** 10.1155/2014/472562

**Published:** 2014-10-15

**Authors:** Kai O. Hensel, Andreas Jenke, Roman Leischik

**Affiliations:** ^1^Department of Cardiology, Faculty of Health, School of Medicine, Witten/Herdecke University, Elberfelderstraße 1, 58095 Hagen, Germany; ^2^HELIOS Children's Hospital Wuppertal, Faculty of Health, Witten/Herdecke University, 42283 Wuppertal, Germany

## Abstract

Early diagnosis of cardiac alterations in hypertensive heart disease is still challenging. Since such patients might have depressed global LV systolic strain or strain rate when EF is still normal, speckle-tracking echocardiography (STE) and tissue-Doppler imaging (TDI) combined with stress echocardiography might improve early diagnosis of cardiac alterations. In this prospective study standard 2D Doppler echocardiography, STE, and TDI were performed at rest and during bicycle exercise in 92 consecutive patients—46 hypertensive subjects with normal ejection fraction and 46 healthy controls. STE and TDI were used to measure global peak systolic LV circumferential strain (CS), longitudinal strain (LS), and longitudinal strain rate (SR). Mean arterial blood pressure was significantly higher in hypertensive patients at rest (100.8 mmHg SD 13.5 mmHg; *P* = 0.002) and during physical exercise testing (124.2 mmHg SD 13.4 mmHg; *P* = 0.003). Hypertensive patients had significantly reduced values of systolic CS (*P* = 0.001), LS (*P* = 0.014), and SR (*P* < 0.001) at rest as well as during physical exercise—CS (*P* < 0.001), LS (*P* < 0.001), and SR (*P* < 0.001). Using STE and TDI, reduced LV systolic strain and strain rate consistent with early cardiac alterations can be detected in patients with arterial hypertension. These findings were evident at rest and markedly pronounced during exercise echocardiography.

## 1. Introduction

Cardiovascular diseases are the most common causes of death in the western world accounting for more than one million of deaths annually in USA [1]. One major risk factor for cardiovascular morbidity and mortality worldwide is arterial hypertension (HTN) [2]. Longstanding HTN leads to left ventricular hypertrophy which has been proven to directly predispose to and aggravate irreversible deterioration of LV function and ultimately results in congestive heart failure [3, 4]. In patients with arterial hypertension, it is therefore crucial to detect LV impairment as early as possible in order to identify patients at high risk for heart failure. Given the high incidence of HTN this is not only important for prognosis, optimal prevention, and treatment strategies in order to improve the overall outcome, but also crucial to reduce the burden of the disease from an economical point of view.

Currently, the gold standard in echocardiographically quantifying systolic left ventricular (LV) function is the ejection fraction (EF) [5]. However, measurement of EF is a simplistic approach, closely correlated to the radial component of myocardial deformation and does not account for the complex three-dimensional deformation with the important longitudinal and circumferential plane. As a consequence, the assessment of EF only detects deterioration in LV function in a relatively advanced state [6]. Subtle changes in myocardial deformation occur prior to the more extensive impairment of the LV that is detectable by changes in EF. In fact, experimental studies of isolated papillary muscles in the setting of chronic pressure overload showed depressed contractility despite normal EF [7–9]. Thus early stages of impaired LV function—when therapeutic intervention might be most beneficial—remain undetected [10].

Strain—defined as myocardial deformation expressed in percent—has been proven to discriminate between hypertensive heart disease, hypertrophic cardiomyopathy, and athletes' hearts [11, 12]. This differentiation is possible because regional deformation may be reduced in hypertrophic cardiomyopathy whereas it may be unchanged or hypercontractile in athletes' hearts. Novel quantitative techniques in echocardiographic deformation imaging such as speckle-tracking imaging (STE) [13] and tissue-Doppler imaging (TDI) [14] can reliably measure LV strain and therefore bare incremental diagnostic potentialities for populations with hypertensive heart disease [15]. STE and TDI make use of different physical techniques in order to measure strain: STE utilizes a two-dimensional greyscale B-mode image [16]. TDI makes use of the well-known Doppler phenomenon. Both STE and TDI have been shown to be more sensitive for detection of subtle changes in LV deformation than EF [17, 18]. TDI was also proven to be useful in order to differentiate between pathologic and physiologic LV hypertrophy [19, 20].

In this study, we tested quantitative deformation stress echocardiography as a diagnostic tool for early detection of cardiac malfunction due to arterial hypertension. We aimed to analyze whether measurement of myocardial strain and strain rate with STE and TDI can detect subclinical LV systolic deformation abnormalities in patients with mild arterial hypertension and preserved EF at rest and during physical stress echocardiography. Subsequently, a direct comparison of the two methods during exercise echocardiography was performed. Depressed strain (rate) values in patients with arterial hypertension might serve clinicians as potential indicators to target preventive diagnostics and potentially the extent of treatment.

## 2. Methods

### 2.1. Patient Characteristics

In this prospective study we examined a total of 92 consecutive subjects (47 women, 45 men; *P* = 0.677) at practice for Cardiology and Sport Medicine in Hagen/Germany. The study group consists of 46 patients (male/female ratio 1.09; mean age 46.6 years; standard deviation (SD) 14.4) with mild arterial hypertension with sufficient treatment for blood pressure (BP) control according to the ACCF/AHA guidelines [21] (Table 1). The control group included 46 healthy controls (male/female ratio 0.84; mean age 44 SD 22.5). Inclusion criteria for patients in the study group were the diagnosis of isolated, essential arterial hypertension for at least 5 years before being included in this study. None of the included patients suffered from end-organ damage such as evidence of renal failure or retinal changes. Diagnosis was made according to the 7th report of the Joint National Committee [21]. Other cardiovascular or noncardiovascular comorbidities that may have an influence on the cardiovascular system such as diabetes mellitus, atrial fibrillation, history of angina pectoris, and coronary artery disease were strict exclusion criteria. Further, a thorough history and physical examination and both resting and exercise echocardiography and EKG were obtained.

Participants in the control group had completely negative medical history with regard to the cardiovascular as well as to any other organ system. Each participant was informed about purpose and procedure of the study and provided a written consent. The study protocol was approved by the Witten/Herdecke University ethics committee.

### 2.2. Standard Echocardiographic Examination

The echocardiographic examination was performed with the commercially available ultrasound device VIVID 7 Dimension by General Electrics (GE) Medical Systems GmbH, Germany. The ultrasound probe used was a “Matrix Array,” Sector 1.5–4.3 MHz. The recorded data was stored and later analyzed with the offline-workstation software EchoPAC Dimension [5.2.0, General Electrics (GE) Medical Systems GmbH, Germany] by two experienced, independent, cardiologists, blinded to both groups.

Transthoracic echocardiography was performed in the left lateral decubitus position. The following measurements with regard to the LV were done in the parasternal long axis and in the apical 4-chamber view: interventricular septum, internal diameter of the cavity, left ventricular posterior wall, aortic diameter, left atrial diameter, left ventricular outflow tract, LV mass, end-systolic volume (ESV), end-diastolic volume (EDV), ejection fraction (EF), stroke volume, and cardiac output. ESV and EDV were calculated with the formula of Simpson.

### 2.3. Speckle-Tracking

Standard cross-sectional 2D images were recorded in the conventional B-mode and afterwards postprocessed on an external offline workstation. For parasternal short axis imaging the transducer was placed in the left parasternal region at the third and fourth intercostal space. The apical views were recorded with the transducer in the fifth intercostal space in the anterior axillary line close to or over the point of maximal impulse. Images were recorded at frame rates between 60 and 80 frames per second for optimal postprocessing measurements. Loops of at least three entire cardiac cycles of the LV were recorded in the cine loop format and synchronized to a 4-Lead ECG. Recordings were performed in a predetermined order using a preset with the following angles to assess the different dimensions of strain. Circumferential strain was measured in the parasternal short axis at the level of the papillary muscles. Longitudinal strain was assessed in the apical 4-, 3- and 2-chamber views. Longitudinal strain rate was detected in the apical 4-chamber view (apical, mid-, and basal septum).

### 2.4. Tissue-Doppler

Three consecutive heart beats were recorded as cine loops using color Doppler imaging at frame rates between 100 and 140 frames per second (fps) and >150 fps for the assessment of strain and strain rate, respectively. The ultrasound beam was aimed parallel to the movement of the myocardium to be analyzed in order to minimize angle deviation. Longitudinal strain was detected at a basal, mid-, and apical level of each wall. Circumferential strain was detected at the septum and free wall. Selection of echo windows and order of image acquisition were identical to those in the procedure for STE.

### 2.5. Stress Echocardiography

After the general echocardiographic studies, speckle-tracking and tissue-Doppler deformation analyses were utilized at three different levels of physical challenge: (1) in the resting state, (2) after cycling at a level of 50 Watts of resistance for two minutes, and (3) at 150 Watts resistance.

Strain and strain rate were assessed in the same procedure as described above with the participant being physically challenged riding a bicycle in a prone position. Cycling resistance was increased by 25 Watts every two minutes. The patients cycled in a prone position and the table was tilted 30 to 45 degrees to the left for image acquisition.

### 2.6. Postprocessing: Assessment of Strain

For speckle-tracking Q-analysis three recorded cardiac cycles were analyzed. The region of interest (ROI) was defined and single points were manually repositioned for optimal tissue-tracking. Strain and strain rate were calculated by the software and presented in a numeric and graphic manner.

For tissue-Doppler strain assessment, exact discrimination of the myocardium is an essential part of the procedure. Similar to the tracking procedure used in STE, the ROI for TDI analysis was defined by the examiner and timed at end-diastole and end-systole. Width and position of the area to be analyzed had to be adjusted manually. The same sample volume was used for all measurements. Angle deviation of the myocardial wall to the Doppler beam was kept to a minimum.

All echocardiographic analyses were performed by the same investigators, who were blinded to the patients' clinical status at the time of assessment of strain and strain rate. The results were reproducible and intra- and interobserver variability were <3.2% in our study.

### 2.7. Statistical Methods

Hemodynamic data and clinical and echocardiographic characteristics of the two groups were described by mean and standard deviation. Box-Whisker Plots were used for the graphic representation of the data distribution. Fisher's exact test was used to compare the two study groups with respect to gender. Hemodynamic data and clinical and echocardiographic characteristics of the two study groups were compared using the Mann-Whitney *U* test. Wilcoxon matched-pairs signed-ranks test was used to compare STE and TDI data. Spearman's rank correlation coefficient and multivariate analysis were utilized to rule out potential confounding of strain (rate) values by differing baseline characteristics. Values for P < 0.05 were considered statistically significant. Stata IC/11.2 for Windows was used for all statistical analysis.

## 3. Results

### 3.1. Epidemiological Characteristics

Epidemiological characteristics including mean age and gender distribution did not differ between the groups except for body-weight-related parameters such as body mass index (28.5 in hypertensive patients versus 23.6 in control patients) (Table 2). Mean arterial blood pressure (BP) was significantly higher in the hypertensive group at rest (100.8 mmHg SD 13.5 mmHg; *P* = 0.002), as well as during exercise testing at 50 Watts (110.7 mmHg SD 11.6 mmHg; *P* < 0.001) and at 150 Watts resistance (124.2 mmHg SD 13.4 mmHg; *P* = 0.003).

Ejection fraction, fractional shortening of the midwall, and E/A-wave ratio were not significantly different between the groups (Table 3). Even though conventional echocardiographic parameters showed minor differences between the groups, parameters were within normal limits according to ASE/AHA recommendations [22, 23]. Conventional exercise echocardiography showed no wall motion abnormalities and exercise EKG was normal in all subjects of both groups. Spearman's rank correlation and multivariate analyses were conducted to rule out LV mass, body weight, BMI, and body surface area as potential confounding factors.

### 3.2. Peak LV Strain Values Are Reduced in Patients with Arterial Hypertension

**Figure 1:**
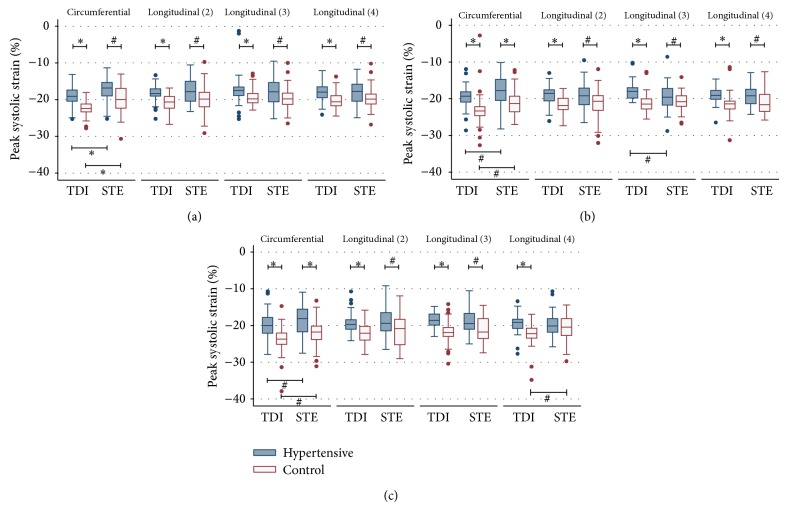
Tissue-Doppler-derived and speckle-tracking-derived circumferential and longitudinal global* strain* of hypertensive patients and healthy controls (a) at rest, (b) at 50 Watts resistance, and (c) at 150 Watts resistance during physical stress echocardiography. TDI: tissue-Doppler imaging; STE: speckle-tracking echocardiography; ^#^ *P* < 0.05; ^*^ *P* < 0.001; *P* values were calculated with the Mann-Whitney-*U* test and Wilcoxon signed-rank test.

Participants with arterial hypertension had lower peak strain values than healthy controls using both speckle-tracking (STE) and tissue-Doppler imaging (TDI) (Figure 1(a)). Specifically, hypertensive subjects showed significantly lower peak LV strain values for circumferential strain (CS) (*P* < 0.001) as well as longitudinal strain (LS) (*P* < 0.001) in the 2-, 3-, and 4-chamber view both at rest (*P* < 0.001) and during exercise testing (*P* < 0.001) (Table 4) (Figures 1(b) and 1(c)). STE- and TDI-derived strain levels showed similar results (Figure 1). At rest healthy participants showed significantly higher strain values in CS (*P* = 0.001) and LS (*P* = 0.014). After cycling at a resistance of 50 Watts for two minutes healthy controls had significantly higher values in CS (*P* < 0.001) and LS in the 2-chamber (*P* = 0.010), in the 3-chamber (*P* = 0.033), and in the 4-chamber view (*P* = 0.011) compared to hypertensive patients. Similar results were found at maximal resistance during exercise echocardiography (Table 5). Subgroup analyses revealed no differences in strain and strain rate between hypertensive patients with and without beta-adrenergic blocking agents (data not shown).

### 3.3. The Increase of LV Peak Strain during Stress Is Impaired in Patients with Arterial Hypertension

The degree of the increase in LV systolic strain was measured using STE and TDI. Hypertensive patients had a weaker increase in peak strain during physical exercise which was detected slightly more distinctively with TDI than with STE. TDI could detect significant increases in CS (*P* = 0.005) and LS (*P* < 0.001) in nine out of sixteen comparisons, mostly in the healthy control group (Supplementary Table 1; Supplementary Material available online at http://dx.doi.org/10.1155/2014/472562). Nonsignificant results showed the same tendency with a stronger increase in strain for the control group. STE analyses showed similar findings (Supplementary Table 2).

### 3.4. Hypertensive Patients Have Reduced Peak Systolic Strain Rate at Rest and during Exercise

**Figure 2:**
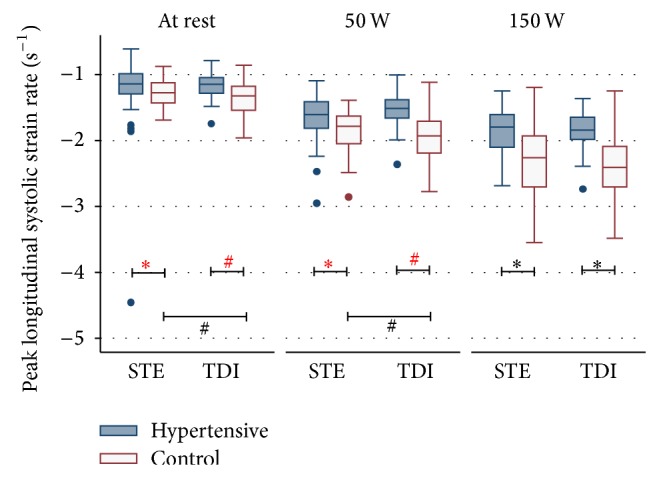
Longitudinal* strain rate* at rest and during physical stress echocardiography in hypertensive patients and healthy controls assessed with tissue-Doppler and speckle-tracking. TDI: tissue-Doppler imaging; STE: speckle-tracking echocardiography; ^#^ *P* < 0.05; ^*^ *P* < 0.001; *P* values were calculated with the Mann-Whitney *U* test and Wilcoxon signed-rank test.

**Figure 3:**
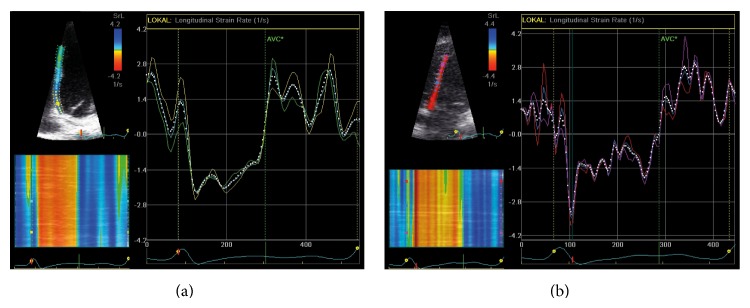
*Speckle-tracking*. Longitudinal* strain rate* at maximal stress during exercise echocardiography (a) in a hypertensive patient and (b) in a healthy control. Note the difference in maximal* strain rate* during early systole.

LV myocardial contractility was measured using STE and TDI. Strain rate was reduced both at rest (*P* < 0.001) as well as during exercise testing (*P* < 0.001) in patients with arterial HTN. This was shown both with STE and TDI. The degree of the increase in LV systolic contractility was higher in the control group (Figures 2 and 3).

## 4. Discussion

### 4.1. LV Myocardial Deformation in Arterial Hypertension

This study demonstrates reduced levels of peak LV systolic peak strain and strain rate at rest in patients with mild arterial hypertension, while conventional echocardiographic parameters such as ejection fraction (EF) and fractional shortening of the midwall (FS) remained unchanged when compared to healthy control patients. Similar findings have recently been shown in an experimental setting for myocardial hypertrophy in rats [24]. In our study, these differences became more pronounced during physical exercise using both speckle-tracking (STE) and tissue-Doppler imaging (TDI). This is in line with the findings of Atilgan et al. who found patients with mild arterial hypertension to have reduced LV longitudinal deformation [25].

Since there were no significant differences in EF and FS between the groups at the moment of investigation, the observed changes most likely reflect cardiac changes due to longstanding arterial hypertension, evident before conventional echocardiographic parameters values become pathologic. It currently remains unclear whether or not these changes can be reversible by adequate blood pressure control, but considering the extent of the observed changes in our study we consider it rather unlikely. Nevertheless, in order to answer this question longitudinal analyses on a large patient cohort with isolated arterial hypertension are necessary.

Furthermore, due to the fact that functional alterations occur inhomogenously in myocardial hypertrophy [26, 27], varying observations for regional versus global systolic strain can be explained. The question of which is the most appropriate aspect of the myocardium for the assessment of strain in this diagnostic content has yet to be addressed.

### 4.2. Quantitative Deformation Analysis during Physical Exercise

Even though stress echocardiography is a powerful diagnostic instrument in the hands of experienced echocardiographers, the value of stress echocardiography as a tool for clinical decision making in myocardial disease is still limited by subjective endocardial border definition and differences in interindividual interpretation of regional wall motion abnormalities. These limitations eventually led to the development of more quantitative methods [28–31] some of which bear true advantages especially for less experienced examiners [32, 33] as, for example, tissue-Doppler imaging which has been showed to be as accurate as visual interpretation by experts [33]. In our study we investigated the value of two quantitative methods, namely, tissue-Doppler (TDI) and speckle-tracking (STE), in assessing mild cardiac abnormalities in hypertensive patients. We could show that hypertensive patients have a less pronounced increase in LV deformation during physical exercise compared to healthy individuals (Figure 1). Both STE and TDI were able to accurately detect peak systolic LV strain values during exercise echocardiography (Tables 4 and 5). Furthermore, the absolute increase in myocardial strain during stress echocardiography (Supplementary Tables 1 and 2) as well as differences in strain rate during exercise testing (Table 6) were significantly different between the two groups as measured by STE and TDI. This first enables STE and TDI to differentiate healthy controls from patients in which strain and strain rate do not increase as much as a result of systolic impairment due to longstanding arterial hypertension. While differences in myocardial contractility were already present at rest in the population we examined in this study, the incremental value of stress testing becomes evident when looking at the development of strain rate during exercise testing. This shows a much more pronounced difference between peak systolic strain rates in hypertensive versus healthy patients at maximal resistance when compared to the resting state (Figure 2). A more subtle change in myocardial strain (rate) might not necessarily become evident at rest and therefore require the addition of STE or TDI during stress testing.

In consequence, both TDI- and STE-derived LV peak strain and strain rate during bicycle stress echocardiography are a useful tool to detect early impairment of myocardial function due to longstanding arterial hypertension. This is supported by data presented by Hanekom et al. who proved that quantitative echocardiography in combination with exercise testing is feasible in an angiographic correlation study [34].

### 4.3. Regional Strain Rate May Underestimate Contractility in the Resting State

Strain rate was measured at rest and during exercise in both groups using STE and TDI at the basal septum only as well as at the entire septum. TDI-derived strain rate values showed a more marked difference between the two groups when compared to STE which becomes less evident at increasing resistance during exercise (Figure 2, Table 6). Interestingly, strain rate values derived locally from the basal septum were less able to distinguish between the hypertensive and healthy hearts when compared to analyzing the entire septum. This effect is stronger for STE and becomes less emphasized at increasing stress levels. Due to these findings, it is therefore important to compare various segments when analyzing strain rate in the resting state, whereas the assessment of a single segment seems to be sufficient during exercise testing. This goes partly in line with the findings of Ingul et al. who showed that mean global (versus regional) strain rate is a good predictor for clinical outcome in patients with abnormal wall motion during stress echocardiography [35].

### 4.4. Study Limitations

In general, despite the promise that quantitative deformation imaging offers, there are still a number of challenges limiting its clinical utility such as time consuming image acquisition, complex methodology, technological requirements, and a lack of consensus regarding optimal parameters and methods [36, 37].

TDI, for example, bears considerable limitations such as angle dependency, the requirement of high frame rates, and the fact that it is prone to artifacts [38, 39]. Misalignment of the insonation angle with the true wall orientation can significantly underestimate strain. While two-dimensional speckle-tracking works on a different technical principle and is therefore an angle-independent method [16, 40], it also has its limitations. For instance, recognized speckles are only detected in a two-dimensional plane, whereas cardiac fibers contract in a three-dimensional manner. Thus, speckles move out of the detectable area. Even though, the software compensates for this by detection of new speckles as they move into the scanned plane [40], low-frame rates still lead to undersampling and speckles move out of the scanned area too soon for digital compensation. High frame rates can reduce this problem but lead to inadequate tracking due to decreased spatial resolution.

## 5. Conclusions

Hypertensive heart disease with normal ejection fraction is associated with reduced peak LV systolic contractility. Speckle-tracking and tissue-Doppler showed depressed LV strain and strain rate in patients with arterial hypertension both at rest and more pronounced during physical stress echocardiography. These findings target strain and strain rate as early parameters for preventive strategies in hypertensive heart disease. Further studies and widespread application of these novel techniques are needed in order to facilitate a more focused utilization and hereby improve their clinical applicability.

## Supplementary Material

In order to assess the increase of myocardial strain during the three phases of physical stress echocardiography, mean differences in peak systolic LV strain were calculated for both STE and TDI-derived strain values. Supplementary table 1 (TDI) and 2 (STE) present the comparison of LV strain values at rest and during exercise testing for both the hypertensive and the control group.
Note the overall lesser increase in myocardial strain in the hypertensive group.

## Figures and Tables

**Table 1:** Antihypertensive therapy regimen in the study group; control subjects were under no influence of antihypertensive medication.

	Hypertensive Patients (*n* = 46)
Behavioural/nutritional management only	14
Beta-Blocker	9
ACE-/AT1-Inhibitor	7
Beta-Blocker and ACE-/AT1-Inhibitor	9
Beta-Blocker, ACE-/AT1-Inhibitor and Ca-Antagonist	7

**Table 2:** Clinical characterisitcs of the entire study population and sub-group analysis, *P*-values: Mann-Whitney-*U*-Test, Fischer's exact test.

		All subjects (*n* = 92)		
		Hypertensive (*n* = 46)	Control (*n* = 46)	*P*-value
	Sex	22 F, 24 M	25 F, 21 M	NS
	Age (years)	46.6 SD 14.4	44 SD 22.5	NS
	Height (cm)	174 SD 10.2	172.9 SD 9.2	NS
	Weight (kg)	86 SD 21.4	70.8 SD 11.9	0.000
	BMI	28.5 SD 7.6	23.6 SD 3	0.000
	BSA (m^2^)	2 SD 0.2	1.8 SD 0.2	0.001

At rest	Systolic BP (mmHg)	131 SD 20.5	121.7 SD 16.7	0.043
	Diastolic BP (mmHg)	85.7 SD 13.5	78.8 SD 10.8	0.011
	Mean BP (mmHg)	100.8 SD 13.5	93.1 SD 10.5	0.002
	Heart rate (bpm)	69.3 SD 11.8	72.4 SD 15.1	NS
	EF (%)	56.3 SD 7.6	57.2 SD 7.2	NS

50 W	Systolic BP (mmHg)	152.0 SD 19.9	147.9 SD 19	NS
	Diastolic BP (mmHg)	90.1 SD 12.2	80.5 SD 11.3	0.000
	Mean BP (mmHg)	110.7 SD 11.6	103 SD 10.2	0.000
	Heart rate (bpm)	107.4 SD 13.2	113.2 SD 16	NS
	EF (%)	58.2 SD 8.1	59.5 SD 8.1	NS

150 W	Systolic BP (mmHg)	185.3 SD 23.4	175.8 SD 22.2	0.016
	Diastolic BP (mmHg)	93.6 SD 11.6	86.4 SD 12.9	0.008
	Mean BP (mmHg)	124.2 SD 13.4	116.2 SD 14.1	0.003
	Heart rate (bpm)	124.5 SD 15.8	140.2 SD 18.1	0.000
	EF (%)	59.8 SD 8.1	61.9 SD 6.6	NS

**Table 3:** General echocardiographic characteristics of the study population, *P*-values were calculated with the Mann-Whitney-*U* test.

	All subjects (*n* = 92)		
	Hypertensive (*n* = 46)	Control (*n* = 46)	*P*-value
Left atrial diameter (cm)	3.1 SD 0.5	2.9 SD 0.5	0.022
Interventricular septal diameter, diastolic (cm)	1 SD 0.2	0.9 SD 0.2	0.016
Interventricular septal diameter, systolic (cm)	1.5 SD 0.2	1.3 SD 0.2	0.020
LV cavity diameter, diastolic (cm)	5.4 SD 0.5	4.3 SD 0.5	0.011
LV cavity diameter, systolic (cm)	3.1 SD 0.5	3 SD 0.5	NS
LV posterior wall diameter, diastolic (cm)	1.1 SD 0.2	1 SD 0.2	NS
LV posterior wall diameter, systolic (cm)	1.5 SD 0.4	1.4 SD 0.2	NS
Fractional shortening of the mid-wall (%)	32 SD 6.6	31.3 SD 5.6	NS
Stroke volume (mL)	57.2 SD 14.5	48.3 SD 15	0.001
Left ventricular mass (g)	188.4 SD 59.4	148.8 SD 44.2	0.001
LV mass/body surface area (g/m^2^)	96.8 SD 30.6	81.1 SD 21.3	0.020
E-Wave/A-Wave	1.2 SD 0.4	1.4 SD 0.5	NS
E-Wave/E^I^-Wave	9.3 SD 2.4	7.4 SD 1.7	0.000
Iso-volumetric contraction time (ms)	79.6 SD 17	72 SD 13.3	0.038
Iso-volumetric relaxation time (ms)	85.5 SD 16.7	84.5 SD 13.7	NS

**Table 4:** Tissue-doppler: peak LV strain at rest and during stress echocardiography.

	All subjects (*n* = 92)		
	Hypertensive (*n* = 46)	Control (*n* = 46)	*P*-value
At rest			
Circumferential	−18.9 SD 2.6	−22.2 SD 2.1	<0.001
Longitudinal (2)	−18.3 SD 2.1	−20.8 SD 2.3	<0.001
Longitudinal (3)	−17.3 SD 4.1	−19.2 SD 2.4	<0.001
Longitudinal (4)	−18.0 SD 2.3	−20.1 SD 2.3	<0.001

50 Watts			
Circumferential	−19.5 SD 3.1	−23.1 SD 4.4	<0.001
Longitudinal (2)	−19.1 SD 2.7	−21.6 SD 2.2	<0.001
Longitudinal (3)	−17.9 SD 2.3	−21.1 SD 2.5	<0.001
Longitudinal (4)	−18.9 SD 2.2	−21.5 SD 3.0	<0.001

150 Watts			
Circumferential	−19.8 SD 3.8	−23.9 SD 3.6	<0.001
Longitudinal (2)	−19.4 SD 2.6	−22.1 SD 2.5	<0.001
Longitudinal (3)	−18.6 SD 2.0	−21.8 SD 3.0	<0.001
Longitudinal (4)	−19.3 SD 2.6	−22.5 SD 2.9	<0.001

**Table 5:** Speckle-tracking: peak LV strain at rest and during stress echocardiography.

	All subjects (*n* = 92)		
	Hypertensive (*n* = 46)	Control (*n* = 46)	*P*-value
At rest			
Circumferential	−17.2 SD 3.0	−19.9 SD 3.7	0.001
Longitudinal (2)	−17.6 SD 3.4	−20.1 SD 3.7	0.002
Longitudinal (3)	−17.9 SD 3.4	−19.8 SD 3.2	0.010
Longitudinal (4)	−18.2 SD 3.3	−19.6 SD 3.0	0.014

50 Watts			
Circumferential	−17.6 SD 4.2	−21.2 SD 3.4	<0.001
Longitudinal (2)	−19.3 SD 3.6	−21.4 SD 3.8	0.010
Longitudinal (3)	−19.5 SD 3.5	−20.9 SD 2.5	0.033
Longitudinal (4)	−19.5 SD 2.9	−21.0 SD 3.1	0.011

150 Watts			
Circumferential	−18.2 SD 4.3	−22.2 SD 3.6	<0.001
Longitudinal (2)	−18.6 SD 4.2	−21.6 SD 4.1	0.005
Longitudinal (3)	−18.9 SD 3.4	−21.0 SD 3.6	0.011
Longitudinal (4)	−19.7 SD 3.3	−20.7 SD 3.4	NS

**Table 6:** Speckle-tracking and tissue-Doppler: Strain rate at rest and during stress testing; *P*-values are calculated with the Mann-Whitney-*U*-test.

	Hypertensive (*n* = 46)	Control (*n* = 46)	*P*-value
At rest			
STE (entire septum)	−1.25 SD 0.57	−1.28 SD 0.21	0.037
STE (basal septum)	−1.22 SD 0.42	−1.17 SD 0.31	NS
TDI (entire septum)	−1.16 SD 0.19	−1.37 SD 0.27	<0.001
TDI (basal septum)	−1.24 SD 0.27	−1.39 SD 0.37	0.030

50 Watts			
STE (entire septum)	−1.65 SD 0.36	−1.86 SD 0.33	0.002
STE (basal septum)	−1.56 SD 0.56	−1.72 SD 0.49	NS
TDI (entire septum)	−1.55 SD 0.25	−2.00 SD 0.39	<0.001
TDI (basal septum)	−1.63 SD 0.30	−2.06 SD 0.42	<0.001

150 Watts			
STE (entire septum)	−1.87 SD 0.37	−2.35 SD 0.53	<0.001
STE (basal septum)	−1.93 SD 0.63	−2.30 SD 0.64	0.004
TDI (entire septum)	−1.85 SD 0.30	−2.42 SD 0.49	<0.001
TDI (basal septum)	−1.85 SD 0.41	−2.55 SD 0.65	<0.001
