# Supplementary material for: Speckle-Tracking and Tissue-Doppler Stress Echocardiography in Arterial Hypertension: A Sensitive Tool for Detection of Subclinical LV Impairment
Source: Biomed Res Int. 2014 Oct 15;2014:472562. doi: 10.1155/2014/472562 (PMC4214170; doi:10.1155/2014/472562)
Supplement: Supplementary file 1 — In order to assess the increase of myocardial strain during the three phases of physical stress echocardiography, mean differences in peak systolic LV strain were calculated for both STE and TDI-derived strain values. Supplementary table 1 (TDI) and 2 (STE) present the comparison of LV strain values at rest and during exercise testing for both the hypertensive and the control group. Note the overall lesser increase in myocardial strain in the hypertensive group. [file 472562.f1.pdf]

	Hypertensive (n=46)	p-value	Control (n=46)	p-value
**Global circumferential strain**				
**Low - rest**	-0.5 SD 4.1	NS	-0.9 SD 4.5	NS
**High - rest**	-0.8 SD 4.5	NS	-1.6 SD 4.1	0.007
**Global longitudinal strain (2-chamber view)**				
**Low - rest**	-0.8 SD 3.4	NS	-0.8 SD 3.3	NS
**High - rest**	-1.1 SD 3.5	0.029	-1.3 SD 3.2	0.014
**Global longitudinal strain (3-chamber view)**				
**Low - rest**	-0.6 SD 4.7	NS	-2.0 SD 3.2	<0.001
**High - rest**	-1.3 SD 4.0	NS	-2.6 SD 3.0	<0.001
**Global longitudinal strain (4-chamber view)**				
**Low - rest**	-0.9 SD 3.1	0.041	-1.5 SD 3.7	0.022
**High - rest**	-1.4 SD 3.2	0.005	-2.4 SD 4.1	<0.001

Supplementary table 1. Tissue-Doppler: Difference in peak LV strain at rest and during stress echocardiography

	Hypertensive (n=46)	p-value	Control (n=46)	p-value
	**Global circumferential strain**			
**Low - rest**	-0.3 SD 5.2	NS	-1.2 SD 4.6	NS
**High - rest**	-1.0 SD 4.5	NS	-2.4 SD 4.8	0.003
	**Global longitudinal strain (2-chamber view)**			
**Low - rest**	-1.7 SD 4.8	0.017	-1.3 SD 5.1	NS
**High - rest**	-0.9 SD 5.4	NS	-1.4 SD 5.6	NS
	**Global longitudinal strain (3-chamber view)**			
**Low - rest**	-1.5 SD 4.3	0.016	-1.1 SD 4.1	0.042
**High - rest**	-1.2 SD 4.2	NS	-1.3 SD 5.0	0.037
	**Global longitudinal strain (4-chamber view)**			
**Low - rest**	-1.2 SD 4.0	0.021	-1.4 SD 4.0	0.012
**High - rest**	-1.6 SD 3.9	0.012	-1.2 SD 4.7	NS

Supplementary table 2. Speckle-tracking: Difference in peak LV strain at rest and during stress echocardiography
